# Environmental Health Education for Asbestos-Contaminated Communities in Italy: The Casale Monferrato Case Study

**DOI:** 10.5334/aogh.2491

**Published:** 2019-06-17

**Authors:** Daniela Marsili, Adriana Canepa, Nicola Mossone, Pietro Comba

**Affiliations:** 1 Department of Environment and Health, Istituto Superiore di Sanità, IT; 2 WHO Collaborating Centre on Environmental Health in Contaminated Sites, IT; 3 Cesare Balbo High School of the Network “ScuoleInsieme”, Casale Monferrato, IT

## Abstract

**Background:**

Environmental health education contributes towards increasing awareness of communities to prevent exposure to hazardous substances. Casale Monferrato, the operating site for the Eternit asbestos-cement factory from 1907 to 1986, is a prioritized asbestos-contaminated site for remediation in Italy. The area is prone to severe asbestos-related diseases. About 50 cases of mesothelioma are diagnosed in Casale Monferrato annually; mesothelioma has been shown to be caused by occupational, environmental and domestic asbestos exposure.

**Objectives:**

The goal of this paper is to analyze the Casale Monferrato case study in terms of youth engagement in environmental health education initiatives on asbestos risk and health impact. The paper aims at underlining the lessons learned in order to share the success of this initiative with other communities living in asbestos-contaminated sites in different countries.

**Methods:**

Peer education methodology has been used through the Asbestos Classroom to involve teachers, students and other local stakeholders in training activities, in selection of the contents for educational materials and interactive tools, as well as in choosing the presentation process for the aforementioned knowledge sharing instruments.

**Findings:**

From November 2014 to June 2018, 185 high school students and teachers were trained through the Asbestos Classroom. Through December 2018, they trained 3,241 classroom visitors. The Classroom relies on an inclusive participative process in which young people play a key role in the network of relationships within their community.

**Conclusions:**

The paper corroborates the importance of engaging the educational system in communication efforts aimed at fostering collective awareness on environmental risk and health-related impacts for communities living in industrially contaminated sites. Considering the global dimension of the asbestos contamination and disease burden, this experience might be of relevance both in countries that banned asbestos and in those where asbestos is not yet prohibited.

## Background

As a process through which people gain more control over their lives, their health and health determinants, environmental health literacy contributes to the empowerment of individuals and communities. This strengthens the ability of individuals and communities to participate in social and environmental health decision-making and actions [1,2]. From an environmental and public health perspective, definitions and scopes of environmental health literacy are continuously evolving, relying on potential contributions for preventing environmentally induced diseases and for empowering individuals and communities to better understand the environmental risks and health-related impacts. This is an essential element for health promotion [3,4], as well as to foster informed participation in decision-making processes.

Environmental health literacy embodies environmental health education (Agency for Toxic Substances and Disease Registry – https://www.atsdr.cdc.gov/environmentaleducation.html, ATSDR website). Environmental health education integrates components of environmental, health and risk education and also supports health promotion, behavioural changes and social actions [5]. In this framework, environmental health education can support individuals and communities to make informed choices for preventing and minimizing exposures to hazardous substances and for improving their health and quality of life [6,7,8,9]. Experiences involving the youth in environmental health education and risk communication in communities living in contaminated sites are not yet systematically addressed by the scientific literature [10], despite polluted areas and the adverse health effects affecting populations living in contaminated areas being environmental and public health issues of global concern [11,12,13,14,15,16]. [Global Alliance on Health and Pollution website http://gahp.net/planning-health-pollution-action-plans/].

The 53 environment and health ministers of the World Health Organization (WHO) European Region have recognised waste and contaminated sites as a priority area for the European policy agenda in the Declaration of the Sixth Ministerial Conference held in Ostrava in 2017 [17]. This result also has been achieved thanks to the initiatives fostered by a European network of institutions engaged in the domain of industrially contaminated sites and health (COST Action – Cooperation in Science and Technology Action “Industrially Contaminated Sites and Health Network” ICSHNet IS1408, website http://www.icshnet.eu/). In this frame, international collaborations have been promoted with the aim of developing a common approach to research as well as common responses to environmental health threats caused by multiple contamination sources.

In Italy, the National Priority Contaminated Sites Legislation adopted in 1998 recognised the town of Casale Monferrato and its neighbouring municipalities as a prioritized contaminated site for remediation because of the presence of the major Italian asbestos-cement factory that was active from 1907 to 1986 [18]. The carcinogenic risk of asbestos is well assessed [19]. The Casale Monferrato site consists of 48 municipalities with a total population of 84,775 (2011 National Census). The town of Casale Monferrato has been experiencing the most severe asbestos-related disease burden, in particular mesothelioma, observed in Italy for several decades. About 50 new cases of mesothelioma are diagnosed annually in Casale Monferrato, a town of 35,000 inhabitants, and most of these cases are associated with individuals who never worked in the Eternit factory but were exposed to asbestos fibres in the environment (the factory by-products were used as insulating material or for paving roads, courtyards and sporting fields) or at home through contaminated work clothes of relatives working in asbestos-cement manufacturing [18].

In order to address the increasing asbestos health impact in Casale Monferrato, specialists from different disciplines adopted an interdisciplinary approach to build and implement an integrated research and care model [20,21]. In this context, the local education system developed a network of schools, *ScuoleInsieme* (“Schools Together”), and the Cesare Balbo High School activated a synergistic collaboration with relevant institutional and social actors (local authorities, environmental and health professionals, social organizations) for environmental and health education and communication on asbestos. In particular, the asbestos issue is the focus of the Asbestos Classroom (Aula Amianto/Asbesto – website http://www.amiantoasbesto.it/parole.html).

The present paper describes the experience of the Asbestos Classroom in Casale Monferrato, highlighting the role of environmental health education in a community living in a severely contaminated site and highlighting the peer education methodology, aimed at engaging students to contribute to increased community resilience. The paper aims to underline the lessons learned to share this successful experience with other communities living in industrially contaminated sites in different countries and settings.

## Methodology

### Methodological Approach

The Asbestos Classroom, which started in November 2014, is characterised by an original methodological approach in the definition of its contents. It has been designed and implemented through collaboration among students, teachers, scientists, artists, journalists and trade unionists, who shared the overarching objective and contributed to define the contents of the dissemination and educational materials and tools. The initiative involves different institutions, such as universities, the Ministry of Education, foundations and the Piedmont Region. The integration of the collected materials in a multimedia interactive tool has been performed by Ecofficina Ltd, a company experienced in creating original multimedia and interactive exhibitions. The goal of the multimedia asbestos classroom was to build a permanent tool to increase knowledge about asbestos risk and health impact, especially among young people, for students of all levels of education. To allow young people to be the protagonists in the management of the Asbestos Classroom, a visual and digital experience was chosen to realize an interactive multimedia tool.

The Asbestos Classroom can be seen as a black box, which places the users at the centre of the experience. The user interacts with the equipped walls and two large touch screens, in a sequence of actions associated with change in colour that reveal hidden symbols and new points of view. This creates awareness of the hazards associated with the use of asbestos and the required remediation actions. As recalled by the project logo, the four basic colours with subtractive synthesis (cyan, magenta, yellow and black), typical of letterpress printing, are the leitmotif of a physical and digital journey in the world of asbestos, encompassing the need to know and the need to go beyond, with the ultimate goal of imagining, and thus enabling, a different future for Casale Monferrato.

### Educational methodology

Peer Education is based on an educational methodology supported since 1997 by the European Union as a way of teaching and learning to realize effective communication [22,23]. In 2000, WHO recommended pursuing this educational methodology to experientially obtain an improvement in student life skills (problem solving, effective communication, understanding, personal and collective effectiveness and the method of continuous learning, learning to learn), as Boda documented in her essays [24,25,26]. This methodology relies on training some members of a group about a theme, usually connected to prevention of risky behavior, to be an educator or tutor for their peer group [22,23].

Since 2006, Cesare Balbo High School has introduced this methodology with training courses for teachers and the realization of projects focused on themes of social and environmental relevance. In the framework of asbestos risk, students and young people are the protagonists of the Asbestos Classroom, and their active participation is essential for the project: first, the students receive specific training through lessons; then, they are able to meet visitors and become trainers by adopting the peer education methodology.

## Results

The results of the Asbestos Classroom can be summarised as follows. High school students and teachers attended training courses through the Asbestos Classroom. Overall, 185 high school students and teachers were trained from November 2014 through June 2018 (see Table 1). The usual schedule of the training course is divided in three parts:

1. Environmental contamination and reclamations in the Casale Monferrato asbestos-contaminated site, usually taken by the local environmental authority.
2. Asbestos-related diseases, research and health care, usually taken by the oncologist and managers of the Zaccheo Hospice in Casale Monferrato and by a representative of the association of asbestos victims and their relatives.
3. Three to four meetings in which the students-already-teachers provide training to other students about the management of interactive and multimedia tools, as well as on the contents of the twelve chapters of the multimedia classroom, including upgrades. These activities are realised with the support of the aforementioned Ecofficina Ltd.

**Table 1:** Number of attendants to the training courses for each category (November 2014–June 2018).

Typology of attendants to the training courses	Number of attendants

Students from high school	117
Teachers from high school	41
Students from high school trained in English	21
Teachers from middle school	6
Total	185

At the end of the course, the Asbestos Classroom trainers can teach different types of visitors. From November 2014 to December 2018, there were 3,241 visitors, including students from 6 to 19 years, teachers, academics, scientists, citizens and association representatives, and special guests (i.e., well-known persons active in different domains, namely science, art, media, policymakers, who have to some extent become testimonials of this project) and tourists visiting Casale Monferrato (see Table 2). In particular, students and teachers from elementary, middle and high schools can visit the Asbestos Classroom. Visits are bookable Cesare Balbo High School’s website (http://www.istitutobalbo.gov.it/?page_id=5947) Asbestos Classroom’s website (http://www.amiantoasbesto.it/).

**Table 2:** Number of visitors of the Asbestos Multimedia Classroom for each category (November 2014–December 2018).

Typology of visitors	Number of visitors

Students from high school	2,370
Students from middle school	264
Students from elementary school	183
Teachers	35
Institutions representatives	72
Associations representatives and citizens	73
Tourists	206
“Special guests”	38
Total	3,241

The activity plan of the Asbestos Classroom also includes the organization of public conferences focused on environmental health, research and reclamations.

In addition, the Asbestos Classroom has been presented at several events, such as the Third Governmental Asbestos Conference (2017) held in Casale Monferrato and the Zero Impact Life event, to show its activities and discuss its educational role. The Zero Impact Life event is organised every year by the Casale Monferrato municipality, associations and schools on Eternot Park, a recreational area built in the location that housed the Eternit asbestos-cement plant before its demolition and complete environmental clean-up. This event aims to show how to live a green life without environmental and health impacts. Since 2016, the event is held during the annual global asbestos awareness week and in memory of the asbestos victims.

The Asbestos Classroom is also fostering the development of new educational projects on environment and health. One on-going project is dedicated to fostering resilience, which is necessary when facing critical situations and for creating effective solutions. Another project focuses on the theme of plastic pollution. The most recent project envisages the English translation of the second section of the Asbestos Classroom (the English version of the first section already exists) and a Spanish translation of the introductory section to promote and support twinning with communities in other countries, namely Colombia and Canada.

The well-acknowledged value of the Asbestos Classroom in Italian and international contexts motivated its inclusion in the European COST Action “Industrially Contaminated Sites and Health Network” (ICSHNet website http://www.icshnet.eu/). As previously mentioned, a COST Action is not a research project, but rather it is a network of research institutions that develop innovative working procedures aimed at responding to societal needs. Since 2015, the ICSHNet represents a collaborative framework aimed at fostering the production of shared documents and other specific deliverables concerning the requirements of environmental and health data, exposure assessment procedures, health impact assessment and risk communication. The Casale Monferrato case study was recognised as part of a risk communication strategy addressed to asbestos-contaminated communities that are still quite numerous in Europe. The Asbestos Classroom was thus presented and discussed at the Annual Plenary Conferences of the Cost Action ICSHNet held in Bonn, February 20–22, 2018, and Rome, February 21–22, 2019 (ICSHNet website http://www.icshnet.eu/). The endorsement of the initiative is motivated by the recognition that it is scientifically founded, technologically advanced and strongly rooted in the community. Based on a multidisciplinary approach, the Asbestos Classroom relies on the active role of young generations, who have the potential for fostering community resilience towards environmental health threats. In the framework of international collaboration activities, the Casale Monferrato municipality and the Italian National Health Institute (*Istituto Superiore di Sanità*), the latter coordinating the COST Action ICSHNet, can promote the sharing of this experience in other European and international settings (see also Adamonyte and Loots) [10].

## Discussion

Networking key stakeholders in communities living in industrially contaminated sites through the engagement of the educational system is an essential action to broaden knowledge and increase awareness, as well as to foster informed participation in the decision-making process concerning risk mitigation and health promotion. The experience in Casale Monferrato represents a success story for a multidisciplinary collaboration aimed at addressing this undertaking. In particular, the network of schools of the Casale Monferrato contaminated site has provided a key contribution to the design and construction of the Asbestos Classroom Amianto/*Asbesto: il coraggio di conoscere/il bisogno di andare oltre”* (“Asbestos: the courage to know/the need to go beyond”) through the adoption of the peer education methodology and the use of interactive multimedia communication tools.

It is worth noting the name of the Asbestos Classroom anticipates the purposes of the initiative: awareness in terms of knowledge and vision for the future. Indeed, the information dissemination and communication materials have been developed taking into account the history of this industrial asbestos-contaminated site, the impact on the territory and the population (living and working environments) as well as the progressive reaction of the Casale Monferrato community towards the burdens of asbestos-related diseases and environmental contamination. An intergenerational dialogue is a key factor for building the collective memory on asbestos within the community, as well as for sharing knowledge on the health impact and awareness for promoting a proactive role of young generations.

The Asbestos Classroom relies on an inclusive participative process in which the students are engaged in the network of relationships within their community. The adoption of the peer education methodology allows the communication and the daily exchange of information with teachers, asbestos ex-exposed workers, patients and their families, health professionals, researchers involved in scientific studies on asbestos impacts in the contaminated area, as well as local authorities. This initiative is playing a key role in promoting the networking of stakeholders and in developing experiences of active citizenship and critical thinking. As a practice of health literacy, this initiative has been contributing to the empowerment of both the individuals and the whole community, representing an effective way for gaining more control over their lives, their health and health determinants.

The adoption of interactive and multimedia communication tools will favour the preservation of the collected information, the histories and the memory of the environmental and health impact of asbestos contamination. Moreover, the use of new Information and Communication Technology (ICT) is facilitating the engagement of young people in formal and non-formal education initiatives on environmental and health issues.

In this frame, it is important to emphasize that the experience of the Asbestos Classroom can be effectively transferred to other communities, both in Italy and globally. There are other experiences in Italy of environmental health education in contaminated sites that have benefited from the collaboration among involved scientists, local authorities, social actors and the educational system. Biancavilla in Sicily is recognised as a site of national priority for remediation due to the presence of the amphibole fluoro-edenite, an amphibolic fibre similar to asbestos, found in a local quarry. Fluoro-edenite contaminated material was extensively used in road paving and the building industry [27]. The Biancavilla prevention model includes communication activities and the engagement of students and teachers. Indeed, health education on fluoro-edenite fibres and exposure modality has been considered as an important formative path for students attending high schools. In particular, M Rapisardi Technological High School has undertaken several initiatives where the students illustrated their schoolwork on fluoro-edenite and related mesothelioma occurrence in Biancavilla during events open to citizens [28].

Another experience of collaborative networking among local authorities, associations of asbestos victims and their relatives and the local educational system is on-going in Emilia-Romagna Region (Northern Italy), in an area previously characterised by the presence of asbestos-cement production that used both chrysotile and crocidolite. In this collaborative frame, students and teachers from Bologna High School “isArt – Liceo Artistico Arcangeli” set up exhibitions on asbestos and wrote an e-book [29], thus increasing awareness of asbestos risk and impact among young people.

Furthermore, it should be stressed that the transferability of the Casale Monferrato experience, which is accessible in an English version, could be of particular value in a wide range of European and international settings.

Notwithstanding a consistent body of evidence about the adverse health effects of asbestos, with the recognition of a special relevance to the causation of pleural mesothelioma responding to both occupational and environmental exposures to this agent (in the absence of other ascertained risk factors), only 65 countries worldwide have so far adopted asbestos bans or severe restrictions, while about 80% of the world population lives in countries where the use of asbestos is still legal (from the International Ban Asbestos Secretariat – IBAS http://www.ibasecretariat.org/). Due to the long latency time of mesothelioma, a remarkable burden of asbestos-related disease may be observed in countries that banned asbestos even several decades after termination of its use [30]. If friable asbestos is not properly removed from dwellings, public buildings and industrial sites, continuous exposure may occur and the end of the current mesothelioma epidemic may be further delayed. It is clear sharing the experience of Casale Monferrato with other communities, which do not yet have access to full and adequate information about the health impact of asbestos and how to prevent exposure, could be particularly valuable.

Asbestos-contaminated communities are generally located close to asbestos quarries or major industries, especially asbestos-cement manufacturing plants, characterised by current and/or past use of asbestos that might still be detectable in the different asbestos matrices (namely soil and air), but also in materials used for the building industry and for paving roads and courtyards. The great variation in exposure patterns and in the time windows considered in available studies corresponds to a variation in the entity and size of observable health impact. Despite this remarkable variation among contexts, consistent patterns in sustaining processes for increasing individual and community resilience have been described in pertinent literature, including environmental and health education. Some of these studies have explicitly mentioned the role of the education system in strengthening the skills of educators, parents and young people themselves [31,32,33,34,35].

Indeed, a key component of community resilience is represented by the ability of the community to actively engage the population, in particular young people [33], in linking individuals to social resources (e.g., networking and social services), as well as in developing capabilities through the enhancement of human capital (healthy and capable population).

## Conclusions

The paper corroborates the importance of engaging the educational system in communication activities aimed at fostering collective awareness on environmental risk and health-related impacts. The successful experience of Casale Monferrato is representative of social community building for empowering individuals and communities living in industrially contaminated sites. The particular attention to young people and their direct involvement in formal and non-formal education initiatives on environmental and public health issues represents good practices in the public health sector. The educational activities described in this paper go beyond simple information dissemination to both young people and the general population. Indeed, networking key stakeholders in a community living in an industrially contaminated site through the engagement of the educational system is an essential action to broaden knowledge, increase awareness and support health promotion. The impact of the environmental educational activities undertaken in Casale Monferrato can be assessed through several indicators. First, the adopted methodology relies on training the trainers: 185 students and teachers were trained to become trainers for 3,241 visitors of the Asbestos Classroom, demonstrating a direct proactive engagement in the initiative. Second, the networking activities involve associations of the asbestos victims and their relatives, trade unions and local authorities because asbestos risk concerns both living and working environments. This significantly influences the perception and awareness of asbestos risk. Third, the long latency time of mesothelioma implies that awareness of the health impact and community preparedness to manage the burden of asbestos-related diseases have to be maintained in the long term.

Taking into account the global dimension of asbestos contamination and disease burden, this experience might be of relevance both in countries that banned asbestos and in those where asbestos is not yet prohibited. The environmental health education initiative presented in this study can be seen as a direct contribution to environmental health literacy aimed at increasing the resilience of affected communities. Environmental health literacy and risk communication are globally recognized actions apt to managing environmental contamination and the related adverse health effects on the population living in contaminated sites. The present paper emphasizes the importance of preserving the histories and the memory of asbestos environmental and health impacts in contaminated communities and the proactive engagement of young people in contributing to community resilience.
